# Targeted therapy with propranolol and metronomic chemotherapy combination: sustained complete response of a relapsing metastatic angiosarcoma

**DOI:** 10.3332/ecancer.2015.499

**Published:** 2015-01-08

**Authors:** Shripad Banavali, Eddy Pasquier, Nicolas Andre

**Affiliations:** 1 Department of Medical and Pediatric Oncology, Tata Memorial Centre, Mumbai 400 012, India; 2 Metronomics Global Health Initiative, Marseille, France; 3 Aix Marseille Université, Inserm, CRO2 UMR_S 91, Marseille 13005, France; 4 Service d’Hématologie & Oncologie Pédiatrique, AP-HM, Marseille 13005, France

**Keywords:** angiosarcoma, metronomic chemotherapy, drug repositioning, palliative treatment, beta-blockers, propranolol, celecoxib, etoposide, cyclophosphamide

## Abstract

We report here a case of a 69-year-old woman with a relapsing metastatic angiosarcoma treated with a combination of metronomic chemotherapy and propranolol. The beta blockers were added since the tumour was positive for betaadrenergic receptor. A complete response was quickly obtained and lasted for 20 months.

With this case, the combination of metronomic chemotherapy and propranolol in angiosarcoma warrants additional studies and illustrates the potential of metronomics to generate innovative yet inexpensive targeted therapies for both high-income and low-/middle-income countries.

## Introduction

Angiosarcomas are rare soft-tissue sarcomas of endothelial origin which have a very poor prognosis [1]. Only a few chemotherapeutics are available for the therapy of relapsing angiosarcoma. Moreover, response rates are typically low, and outcomes are rapidly unfavourable. Recent trials with targeted agents reported limited efficacy [1] except for Avastin [2], which is unaffordable for most patients in a global oncology context [3]. Herein, we report a long-term complete response obtained for the treatment with a combination of metronomic chemotherapy (MC) and the non-selective β-blocker propranolol.

A 69-year-old female presented in March 2011 with a large lesion and few satellite lesions on a lymphedematous left extremity as she had been previously 12 years ago treated for a locally advanced ER/PR negative infiltrating duct carcinoma of the breast with three cycles of cyclophosphamide–adriamycin–5fluorouracil (CAF), breast conservative surgery followed by another three cycles of CAF chemotherapy, radiation therapy, and tamoxifen for 5 years.

**Figure 1.**
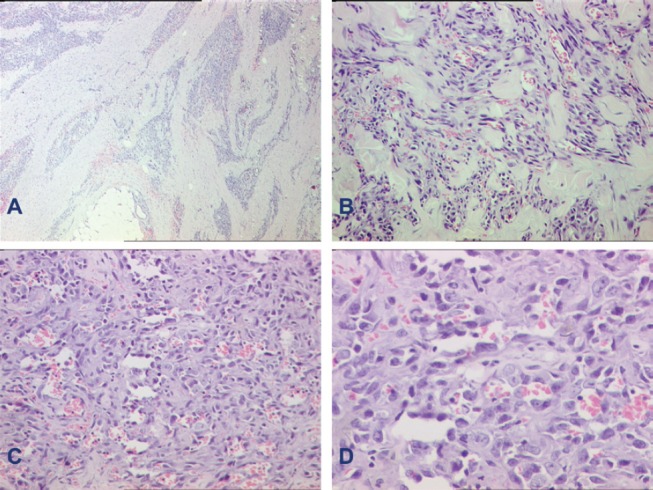
Histomorphology of specimen. A: Photomicrograph of cutaneous angiosarcoma at low power showing an infiltrative tumour. B: Higher power shows tumour cells lining vascular channels. C: Another photo showing the marked cytological atypia in the tumour cells lining vascular channels. D: Another higher power to highlight the cytological atypia of these vasoformative tumour cells. (A—haematoxylin-eosin, X40; B and C—X200; D—X400.)

Pathological analysis of a tumour biopsy led to the diagnosis of angiosarcoma (Figure 1). Imaging work-up did not reveal any metastasis. She underwent forequarter amputation of the left upper extremity in April 2011. The histopathology report confirmed microscopic resection.

In June 2011, the patient was diagnosed with both recurrent local and metastatic disease. Diagnosis and prognosis were discussed with the patient. The patient refused any injectable chemotherapy and was proposed a combination of oral MC and drug repositioning [3] with daily celecoxib 200 mg bid for 3 months along with etoposide 50 mg and cyclophosphamide 50 mg daily for 21 days of a 28-day cycle for 6 months and 15 days of a 28-day cycle for another 6 months. Propranolol 40 mg bid was added because the tumour was positive for beta-adrenergic receptors on RT-PCR.

A complete clinical response was observed after two cycles of therapy. Treatment was gradually tapered down. This three-drug treatment was then given for 1 year and at last only propranolol 20 mg bid and cyclophosphamide 50 mg was given on alternate days for another 6 months. No grade III or IV toxicities were observed. All treatments were stopped in January 2013.

The patient relapsed (left anterior and lateral chest wall; and small lung nodule) 20 months after initiation of the metronomic treatment and was treated with local palliative radiotherapy and oral thalidomide 100 mg with some response and ultimately died of progressive disease in September 2013, 27 months after first relapse.

Relapsing/metastatic angiosarcoma remains a therapeutic challenge as standard MTD chemotherapy, and targeted therapies have so far led to very limited responses rate and overall survival [1, 2].

Interestingly, MC has been reported to induce sustained control of relapsing angiosarcoma in case reports [4, 5] or small series of patients [6, 7]. Thus Vogt *et al* [6] set a pilot study of a multidrug metronomic combination with low-dose trophosphamide, the peroxisome proliferator-activated receptor gamma agonist, pioglitazone, and the selective cyclooxygenase-2 inhibitor, rofecoxib in six patients with advanced malignant vascular tumours. They reported two complete remissions, one partial remission and three stable diseases with a median progression-free survival of 7.7 months. Elsewhere, Mir *et al* [7] reported that single agent metronomic cyclophosphamide led to one complete response (hepatic epithelioid haemangio-endothelioma) and five partial responses in patients with radiation-induced sarcomas with a median progression-free survival of 7.8 months. MC has therefore very likely contributed to the long-term control of the tumour observed in the patient we report here. Because Avastin has also been reported to have a significant clinical activity in angiosarcoma [3], one might speculate that clinical activity of MC is related, at least in part, to its antiangiogenic properties [8].

Beta-blockers have recently been reported to have anticancer effects in several tumours [9]. Additionally, the potential anticancer effect of β-blockers on angiosarcoma *in vivo* and *in vitro* has recently been published [10], and reports have shown the significant expression of beta-adrenergic receptors in several types of vascular tumours [11] which was confirmed using PCR on tumour samples from our patient. Finally, Pasquier *et al* [12, 13] have reported a synergy between chemotherapy and β-blockers in breast cancer or neuroblastoma. Altogether, these findings suggest that propranolol may have contributed to the complete and sustained clinical response observed in this patient.

## Conclusion

With this case, the combination of MC and propranolol in angiosarcoma warrants additional studies. This observation also illustrates the potential of metronomics to generate innovative yet inexpensive targeted therapies [8] for both high-income and low-/middle-income countries [3].
